# Worries and Concerns among Inflammatory Bowel Disease Patients Followed Prospectively over One Year

**DOI:** 10.1155/2011/492034

**Published:** 2011-09-06

**Authors:** Lars-Petter Jelsness-Jørgensen, Bjørn Moum, Tomm Bernklev

**Affiliations:** ^1^Oslo University Hospital Aker, Oslo, Norway; ^2^Østfold Hospital Trust, Cicignongt 6, 1606 Fredrikstad, Norway; ^3^Institute of Clinical Medicine, Faculty of Medicine, University of Oslo, Trondheimsveien 235, 0528 Oslo, Norway; ^4^Oslo University Hospital, Trondheimsveien 235, 0528 Oslo, Norway; ^5^Telemark Hospital, Ulefossveien, 3710 Skien, Norway

## Abstract

Disease-related worries are frequently reported in inflammatory bowel disease (IBD), but longitudinal assessments of these worries are scarce. In the present study, patients completed the rating form of IBD patient concerns (RFIPC) at three occasions during one year. One-way analysis of variance (ANO VA), *t*-tests, bivariate correlation, and linear regression analyses were used to analyse data. The validity and reliability of the Norwegian RFIPC was tested. A total of 140 patients were included (V1), ulcerative colitis (UC) *n* = 92, Crohn's disease (CD) *n* = 48, mean age 46.9 and 40.0-year old, respectively. The highest rated worries included having an ostomy bag, loss of bowel control, and reduced energy levels. Symptoms were positively associated with more worries. A pattern of IBD-related worries was consistent over a period of one year. Worries about undergoing surgery or having an ostomy bag seemed to persist even when symptoms improved. The Norwegian RFIPC is valid and reliable.

## 1. Introduction

In inflammatory bowel disease (IBD), comprising Crohn's disease (CD) and ulcerative colitis (UC), the measurement of health-related quality of life (HRQOL) has become important, both as a primary and secondary endpoint [1–4]. In IBD, the decrease in HRQOL scores is well documented in a vast amount of studies [2–4]. Subjective health measurements in patient research may reveal important issues for the patient, but not apparent for the healthcare worker [1].

In accordance with observations made in clinical practice and with intention to help clinicians quantify information about IBD- related worries, Drossman et al. [5] developed the rating form of IBD patient concerns (RFIPC). Various studies have made use of the RFIPC in clinical trials [5–10]. These studies do, however, have a cross-sectional design, which only provide a snapshot at a given point of time. Since worries most often is future directed, they may potentially change in time and space. Our knowledge of IBD-related worries in a longitudinal perspective is limited. Only one study addresses these issues prospectively in CD, but not in UC [11]. Results of a longitudinal assessment of IBD-related worries may facilitate patient-physician communication and consequently be clinically impactful [1]. 

Different studies have found the RFIPC to be valid and reliable [5–10]. However, the RFIPC needs to be translated and tested psychometrically in the Norwegian language to overcome conceptual, semantic, and linguistic differences across cultures and languages [1, 12].

 The primary aim of this study was to assess disease-related worries and concerns among IBD patients followed prospectively for one year. Secondarily, we aimed to investigate whether IBD-related worries was associated with Sociodemographic and clinical data. In addition, we wanted to evaluate the psychometric properties of the Norwegian version of the RFIPC.

## 2. Materials and Methods

### 2.1. Subjects

Participants were consecutively recruited from three outpatient clinics in south-eastern Norway (the counties of Østfold and Hedmark) during routine follow-up visits. Outpatient clinics usually get referred patients that have poor disease control and need stabilisation, such as for example, UC patients that are not maintaining remission on 5-ASA or CD patients that have relapsed without any current medical treatment. Patients treated with, for example, azathioprine (AZA) or biologics, such as infliximab (remicade) or adalimumab (humira) are all followed by gastroenterologists at outpatient clinics. Participants recruited into this study who had to be aged 18 years old or above, with previously verified IBD, either clinically, endoscopically, or histologically, and without severe disease activity (defined as Simple (SCCAI clinical colitis activity index) [13] or Crohn's (Simple SCDAI disease activity index) [14] score under 10) were eligible for inclusion in this study. The latter since the aim of this study was solely to measure disease-related worries in patients whose condition had stabilized into either remission, mild, or moderate disease activity. Patients were excluded if they had cognitive impairment and were deemed unlikely to comply with the study procedures or if they participated in another study. At each centre, a consultant gastroenterologist was in charge of the study protocol. The inclusion period was from ultimo 2005 to primo 2007. Sociodemographic, clinical, and patient reported outcome (PRO) measures were collected from all patients at baseline (V1) and after 1 year (V3). In order to examine the test-retest reliability of the RFIPC, patients from two out of the three clinics (*n* = 71) were invited to fill out the questionnaires a second time after 6 months (V2).

### 2.2. Clinical and Sociodemographic Data

Sociodemographic variables were gathered by interview, and data regarding clinical status and symptoms were obtained through laboratory tests, medical records, and disease activity indices (SCCAI/SCDAI). In addition, we asked the patients to complete a symptom-based questionnaire that graded their self-perceived IBD symptoms during the previous 14 days [3], using the following categories: no symptoms, mild symptoms (did not interfere with everyday activities), moderate symptoms (interfered with everyday activities and may have resulted in sick leave), and severe symptoms (unable to perform everyday activities, on sick leave or hospitalised). 

Each patient's phenotype was classified according to the Vienna classification for CD patients, as the Montreal classification did not exist when the study protocol was designed. The UC patients were classified into three subgroups: proctitis, left-sided colitis (with inflammation up to the splenic flexure), and extensive colitis (with inflammation beyond the splenic flexure).

## 3. Questionnaires

### 3.1. The Rating Form of Inflammatory Bowel Disease Patient Concerns (RFIPC)

The RFIPC is a disease-specific questionnaires developed by Drossman et al. [5]. This questionnaire rates various important worries and concerns that are raised by IBD patients. The questionnaire consists of the 25 most frequently reported concerns reported by IBD patients, with every item framed in the same style, for example, “Because of your condition, how concerned are you with …?” The responses were scored on a 100-mm horizontal visual analogue scale (VAS). A score of 0-mm represents no worries/concerns, and a score of 100-mm represents the highest possible worries and concerns. The mean score of all 25 items yields the “sum score”.

### 3.2. Translation of the RFIPC Questionnaire

The forward and backward translation was performed following general guidelines [15, 16]. The RFIPC was translated from American English to Norwegian by two independent translators, one gastroenterologist and one clinical research scientist, both fluent in English and with Norwegian as their native tongue. After the two translations had been compared, a preliminary version was agreed upon. The Questionnaire was then translated back into English by a professional translator whose native language was English and who was unfamiliar with the background objectives of the study. This translation was then compared to the original version, and a final version of the questionnaire was agreed upon.

### 3.3. Short Form-36 (SF-36)

The Short Form-36 (SF-36) is a generic, self-administered questionnaire containing 36 items [17] that are divided into 8 multi-item scales consisting of physical functioning (PF), role physical (RP), bodily pain (BP), general health (GH), vitality (VT), social functioning (SF), role emotional (RE), and mental health (MH). For each question, the raw score was coded and transformed into a scale from 0 to 100, with 0 and 100 representing the lowest and highest level of function, respectively. The SF-36 has been validated by others [18].

### 3.4. Inflammatory Bowel Disease Questionnaire (N-IBDQ)

The IBDQ is a disease-specific questionnaire developed by Irvine et al. [19, 20]. The original version consists of 32 items that are divided into four dimensions: bowel symptoms (e.g., loose stools or abdominal pain), systemic symptoms (e.g., fatigue or altered sleep patterns), social function (e.g., work attendance), and emotional function (e.g., anger or depression). The Norwegian validation study (N-IBDQ) revealed a five-dimensional structure: emotional function-1 (EF-1) (fatigue, energy), bowel function-1 (B1) (stool consistency and pattern), bowel function-2 (B2) (bowel pain and discomfort), social function (SF) (work attendance, cancelling social events), and emotional function-2 (E2) (Worries) [4]. All of the responses were scored on a 7-point Likert scale, with a score of 7 representing no problems and a score of 1 representing severe problems. This gives a possible score range of 32–224, with higher scores reflecting an improved HRQOL [4, 19, 20].

## 4. Statistical Methods

Descriptive analyses were performed using descriptive statistics, *t*-test and one-way ANOVA. Associations between clinical/epidemiological data and RFIPC scores were tested with a *t*-test for binary variables; otherwise, bivariate correlation analyses were used. Factors significantly associated with RFIPC outcome were entered into a linear regression analysis.

The validity and reliability of the Norwegian RFIPC was tested following recommendations in the literature [1, 21].

The responsiveness of the RFIPC was calculated through the change of perceived IBD symptoms [5] from V1 to V2. Both a paired *t*-test and Cohen's *d* were used. Scores of 0.2, 0.5, and 0.8 were regarded as small, medium, and large effect sizes, respectively [22].

All tests were two-sided and used a 5% significance level. All data was analysed using SPSS v.17 and v.18 (IBM Inc, Somers, US).

## 5. Ethical Considerations

The study was performed in accordance with the principles of the Helsinki declaration, and approval was obtained from the Regional Ethics Committee and the Norwegian Data Inspectorate before the study was commenced.

## 6. Results

One hundred and forty four patients diagnosed with either UC or CD gave written informed consent for participation in the study. One patient was excluded due to severe disease activity at inclusion (SCDAI > 10), one patient withdrew from the study after a few weeks, and two patients were excluded due to incomplete responses to the questionnaires. A total of 140 patients (UC, *n* = 92; CD, *n* = 48) had sufficient data for statistical analysis at the baseline. Of patients invited to attend the 6 months (V2) and one year (V3) follow-up visits; 63 out of 71 (UC, *n* = 42; CD, *n* = 21) and 133 out of 140 (UC, *n* = 86; CD, *n* = 47) participated, respectively. At V2 and V3, all patients had sufficient data for statistical analysis.

### 6.1. Sociodemographic and Clinical Data

Primary Sociodemographic and clinical data at study entry are presented in Table 1. The mean SCCAI for UC patients was 2.79 (SD 2.39) and 2.35 (SD 2.40) at V1 and V3, respectively. The comparable number in SCDAI for CD patients was 3.9 (SD 2.7) and 3.8 (SD 3.0) at V1 and V3, respectively. In the last year prior to entry, the number of UC and CD patients who had experienced a relapse was 57% and 50%, respectively. The disease activity indices did not differ significantly from V1 to V3 in UC or CD patients (*P* = 0.22 and *P* = 0.86, respectively). Moreover, there were no significant differences in patients reporting no, mild, moderate, or severe IBD symptoms from V1 to V3. In UC however, there were numerically more women than men who reported their IBD symptoms to be improved from V1 to V3, but these differences were not significant.

### 6.2. Scale Statistics and Impact of Clinical/Epidemiological Data

The mean (SD) RFIPC sum score was 29.0 (17.3) and 27.2 (19.5) for UC patients at V1 and V3, respectively. The comparable numbers for CD patients were 32.2 (18.8) and 32.4 (21.9). There were no significant differences in the RFIPC sum score for the UC or CD patients at baseline (V1) or after one year (V3). The CD patients had numerically higher mean RFIPC sum scores than UC patients at all visits, but these differences were not statistically significant. The numerical ranking of individual RFIPC items at V1 and V3 is presented in Table 2. The change in numerical rankings of RFIPC items from V1 to V3 was marginal for both UC and CD patients. Worries about “having an ostomy bag, energy level, loss of bowel control, passing the disease on to the children and developing cancer” were the five worries rated as most important regardless of diagnosis and visit number. In addition, worries about “being treated as different” and “ability to have children” were ranked least important at all visits for both UC and CD, with floor effects of 16.9 and 30.3%, respectively. “Ability to have children” was associated with younger age and the female gender. There were no significant differences in mean RFIPC sum score at neither V1 nor V3 between patients who either had or had not experienced a relapse in the year prior to entry, regardless of diagnosis. The numerical ranking of RFIPC items was also similar in this respect. When differentiating between patients reporting no, mild, moderate, and severe IBD symptoms, the patterns of worries were almost identical for both UC and CD. However, numerical scores on the RFIPC VAS increased successively with increased symptoms (Table 3).

In CD patients, we did not find any associations between the RFIPC sum scores and Sociodemographic and clinical data neither at V1 or V3. In UC as in CD, there were limited associations between Sociodemographic, clinical data and RFIPC sum scores. Increasing IBD symptoms was found to be the only factor of influence on RFIPC sum scores among UC patients at both V1 and V3 (*P* < 0.01).

### 6.3. Validity, Reliability, and Responsiveness of the RFIPC

Factor analysis (maximum likelihood method) performed on the RFIPC items at baseline resulted in the extraction of six factors explaining 70% of the total variance. This was also reproduced when analysing each diagnosis separately. Factor loadings are presented in Table 4.

Spearman's correlation coefficients were calculated between the RFIPC subscales and the domains of N-IBDQ and SF-36, respectively. As expected, we found moderate-to-high correlations between the RFIPC and the psychological, social, and mental domains of N-IBDQ/SF-36. The correlations were generally lowest in the physical HRQOL dimensions. 

The Cronbach's alpha coefficient, which measures internal consistency reliability, was found to be 0.94, indicating that the intercorrelation of items in the RFIPC was very good and consequently measured the same construct. There were only eight and fourteen patients in the CD and UC group, respectively, who reported their condition to be unchanged from V1 to V2. Thus the test-retest reliability results are presented together in Table 5, as the groups showed a similar pattern. The intraclass correlation coefficient (ICC) is used to quantify the degree to which individual RFIPC scores at V1 and V2 resemble each other. A score of 0 and 1 represents no and high reliability, respectively. We found ICC's in our study indicating high reliability.

Twenty-two patients reported their IBD condition to be unchanged at V2. Because the same pattern of responsiveness was observed when analysing UC and CD patients separately (data not shown), we chose to report the data together in Table 6. Overall, responsiveness of the RFIPC was good.

## 7. Discussion

IBD is characterised by symptoms, such as frequent bowel movements, abdominal pain, and rectal bleeding, that cycle between remission and exacerbation. These symptoms may, in conjunction with psychosocial factors, result in secondary consequences such as impaired health-related quality of life (HRQOL) and increased fatigue [2–4, 23]. 

Even though, worries can be regarded as a normal feature of life, we are frequently confronted with IBD-related worries and concerns reported by patients in clinical practice. The RFIPC was developed to help clinicians obtain and quantify this kind of information [5]. This is the first study to report the worries and concerns of both UC and CD patients in a prospective, longitudinal perspective. Blondel-Kucharski et al. [11] used the RFIPC to study worries of CD patients followed for one year. However, they included both in- and outpatients, and, consequently, the participants are quite heterogeneous. In the present study, we included only outpatients without severe disease. Our findings indicate that the pattern of worries in IBD is relatively consistent over a period of one year However, the level of worries, as measured with the RFIPC, increased successively with IBD symptoms. The level of worrying as well as the numerical ranking of worries seem also to be unrelated to having experienced a relapse in the past year. These findings may indicate that it is the current IBD symptoms that are important for the level of worrying and not a history of serious events in the past. The pattern of worries found in our study also seems to be in accordance with the findings of cross-sectional studies that have used the RFIPC [5–11]. 

The potential need of having an ostomy bag was rated as the most important worry by UC patients. A possible explanation may be linked to the dramatic consequence that an ostomy bag may have on daily living and body image. In CD, loss of bowel control was rated as most important. At the opposite end of the spectrum, “being treated as different” and “the ability to have children” were rated least important at all of the visits, regardless of diagnosis. Having a chronic condition like IBD may potentially result in a wide range of environmental responses. However, our findings indicate that neither UC nor CD patients are particularly worried that they will be stigmatised by their social environments. Similar findings have been reported in other studies [5, 6]. Fertility may be influenced by disease activity, surgery, and medications [24]. There is also a risk of adverse events during pregnancy [24]. However, we did find that the “ability to have children” is rated least important among all 25 RFIPC items. One possible explanation may be that this item is unsuitable for use in studies covering a wide range of age groups, because this issue probably is of greater concern to patients in their twenties than to patients in their sixties. Indeed, this is underlined by the fact that more worries of this item were associated with both a younger age and the female gender. 

The only factor in UC patients that seems to be associated with higher levels of worry over time is the presence of IBD symptoms. Our findings indicate that worries are closely correlated with the disease course. This causality is supported by the test-retest analysis between V1 and V2. Worries related to undergoing surgery or having an ostomy bag (factor six) seems to be relatively consistent even when IBD symptoms improve. However, worries do increase when the IBD condition deteriorates [5, 11]. However, Hjortswang et al. [7] reported weak correlations between the number of daily stools and overall RFIPC outcome in his study of Swedish UC patients. In CD patients, we were not able to predict RFIPC outcome at V1 or V3, which may be a result of type 2 statistical errors.

In the RFIPC, the number of factors differs across various studies; in addition, there are discrepancies in the RFIPC items that relate to various factors [5–11]. We found 6 factors that add further complexity to the scaling of the RFIPC. In particular, these variations limit the possibility of comparison across studies and, potentially, the use of RFIPC in clinical trials. Only one of our factors (Factor four; intimacy) was identical to a factor in the original validation by Drossman et al. [5] (Factor two; sexual intimacy). The need of standardisation in health-related quality of life questionnaires has also been put forward in a recent review [25]. The use of the same questionnaires in different studies does not necessarily guarantee comparable results due to different questionnaire versions, translations, and methods of computing and presenting the data. 

 One possible solution to the problem of comparability is to use the RFIPC sum score of all 25 items. This method has been applied in several studies using the RFIPC [26]. We found mean sum scores at baseline to be lower than those reported by Drossman et al. [5], but higher than the results from studies performed in Sweden [6, 7]. However, the wide use of sum scores for the comparison of RFIPC results has been criticised [26]. The sum score implies that one considers specific worries and concerns to be part of one general underlying dimension, which is debateable [26]. Another useful method is to report the results of the individual RFIPC items, as we do in this study. To be able to address IBD-related worries in clinical practice, it is essential to know specifically what patients worry about. 

The reliability of the RFIPC was found to be high, with an ICC of 0.98 (RFIPC sum score) and a Cronbach's alpha of 0.94; this is in accordance with previous validations [5–7]. Our findings also indicate that the RFIPC is able to detect changes over time and, consequently, is suitable for use in longitudinal evaluations [27]. 

In conclusion, patients with IBD seem to experience the same pattern of disease-related worries when followed prospectively over a one-year period. Higher levels of worries are associated with increased IBD symptoms. In addition, worries about undergoing surgery or having an ostomy bag seem to persist even when IBD symptoms improve. Finally, we have demonstrated the Norwegian RFIPC to be valid and reliable.

##  Disclosure 

The authors of this paper declare that this paper is submitted solely to Gastroenterology Research and Practice.

##  Conflict of Interests

The authors declare no conflict of interests.

## Figures and Tables

**Table 1:** Primary Sociodemographic and clinical data at study entry (V1).

	UC (*n* = 92)	CD (*n* = 48)
Age (mean) (SD)	46.9 (5.8)	40.0 (15.0)
Age range	20–82	19–69
Gender		
Female	43 (47)	36 (75)
Male	49 (53)	12 (25)
Educational level		
Second level, first stage (lower)	16 (17.4)	5 (10.4)
Second level, second stage (medium)	42 (45.7)	22 (45.8)
Third level (university)	34 (37.0)	21 (43.8)
Smoking (yes)	23	20
Civil status		
Single	15 (16.3)	10 (21)
Married/cohabitant	70 (76.1)	34 (71)
Divorced/separated	3 (3.3)	3 (6)
Widow/widower	4 (4.3)	1 (2)
Disease duration yrs mean (SD)	8.5 (9.5)	9.2 (9.6)

Abbreviations: UC: ulcerative colitis, CD: Crohn's disease. Figures are in numbers and (%), if not otherwise noted.

**Table 2:** Numerical ranking of disease-related worries and concerns among IBD patients followed for 1-Year (items ranked as the five most important are in boldface).

RFIPC item	UC		CD	
	V1 (*n* = 92)	V3 (*n* = 86)	V1 (*n* = 48)	V3 (*n* = 47)
Financial difficulties	18	18	17	23
Pain and suffering	16	13	12	12
Ability to achieve full potential	10	7	8	8
Loss of bowel control	**5**	**3**	**2**	**1**
Developing cancer	**2**	**2**	**4**	**6**
Dying early	12	10	7	7
Being a burden on others	6	**5**	9	**3**
Attractiveness	21	16	18	17
Feeling alone	22	19	23	22
Feeling out of control	11	9	10	9
Feeling dirty and smelly	19	21	16	16
Ability to perform sexually	20	22	21	19
Ability to have children	25	25	25	25
Passing the disease on to your children	**3**	**4**	**5**	**4**
Being treated as different	24	24	24	24
Having surgery	8	12	6	11
Having an ostomy bag	**1**	**1**	**1**	**5**
Producing unpleasant odors	13	20	14	14
Energy level	**4**	6	**3**	**2**
Feelings about my body	15	15	19	15
Intimacy	23	23	22	21
Loss of sexual drive	17	17	15	18
Having access to quality medical care	14	14	20	20
Uncertain nature of my disease	7	8	11	13
Effects of medication	9	11	13	10

Abbreviations IBD: inflammatory bowel disease, V1: visit 1 (baseline), V3: visit 3 (1-Year), RFIPC: rating form of inflammatory bowel disease patient concerns, UC: ulcerative colitis, and CD: Crohn's disease.

**Table 3:** Determination of the RFIPC's ability to discriminate between IBD symptom severity scores at baseline.

	UC (*n* = 92)				
	None	Mild	Moderate/severe	*F*-value	*P*-value
RFIPC dimensions					
F1 Impact of disease	15.5 (12.6)	29.1 (20.0)	43.2 (18.8)	14.895	<0.001
F2 Expectancy	23.5 (17.1)	40.6 (26.0)	44.6 (24.9)	5.584	0.005
F3 Treatment	15.7 (11.2)	34.5 (23.1)	42.6 (25.7)	10.082	<0.001
F4 Intimacy	9.4 (9.6)	21.4 (17.4)	28.1 (24.4)	6.723	0.002
F5 Stigma	11.0 (13.0)	21.1 (22.1)	29.6 (22.7)	5.205	0.007
F6 Complications	15.8 (15.1)	30.2 (15.6)	38.0 (17.8)	4.142	0.019
Sum score	16.0 (11.3)	30.2 (15.6)	38.0 (17.8)	13.165	<0.001

	CD (*n* = 48)				
	None	Mild	Moderate/severe	*F*-value	*P*-value

RFIPC dimensions					
F1 Impact of disease	28.5 (25.1)	31.7 (19.0)	44.6 (19.1)	2.633	NS
F2 Expectancy	38.8 (36.4)	41.4 (26.7)	44.2 (29.1)	.102	NS
F3 Treatment	34.3 (33.3)	28.9 (18.6)	30.7 (22.4)	.176	NS
F4 Intimacy	19.1 (28.1)	20.6 (19.9)	27.2 (26.7)	.489	NS
F5 Stigma	16.8 (29.4)	26.4 (23.6)	21.2 (19.2)	.597	NS
F6 Complications	28.0 (28.7)	30.8 (20.8)	23.1 (20.7)	.572	NS
Sum score	28.6 (26.3)	31.4 (16.6)	35.1 (18.8)	.377	NS

Abbreviations: RFIPC: rating form of inflammatory bowel disease patient concerns, F1 : F6 (Factors 1–6), UC: ulcerative colitis, CD: Crohn's disease. Figures are presented as means and standard deviations.

**Table 4:** Factor analysis of the RFIPC using maximum likelihood method.

		Factor 1	Factor 2	Factor 3	Factor 4	Factor 5	Factor 6
	*Impact of disease*						
(1)	Financial difficulties	0.59					
(2)	Pain or suffering	0.60					
(3)	Ability to achieve full potential	0.68					
(4)	Loss of bowel control	0.46					
(9)	Feeling alone	0.47					
(10)	Feeling out of control	0.53					
(19)	Energy level	0.71					
	*Expectancy*						
(5)	Developing cancer		0.72				
(6)	Dying early		0.82				
(7)	Being a burden on others		0.48				
(14)	Passing the disease to your children		0.50				
	*Treatment*						
(23)	Having access to quality medical care			0.66			
(24)	Uncertain nature of my disease			0.73			
(25)	Effects of medication			0.74			
	*Intimacy*						
(12)	Ability to perform sexually				0.63		
(21)	Intimacy				0.86		
(22)	Loss of sexual drive				0.64		
	*Stigma*						
(11)	Feeling dirty or smelly					0.74	
(18)	Producing unpleasant odors					0.77	
(15)	Being treated as different					0.41	
	*Complications*						
(16)	Having surgery						0.61
(17)	Having an ostomy bag						0.92
(8)	Attractiveness	(0.34)					
(13)	Ability to have children				(0.16)		
(20)	Feelings about my body	(0.39)					

**Table 5:** Test-retest reliability with intraclass correlation coefficients (ICC) in patients reporting no change in their condition from baseline to followup after 6 months.

	V1 mean (SD)	V2 mean (SD)	Mean Difference (95% CI)	ICC
RFIPC factors				
Impact of disease	37.2 (22.4)	35.8 (22.0)	1.4	.90
Expectancy	48.2 (29.3)	44.8 (29.1)	3.4	.88
Treatment	30.2 (27.6)	29.3 (25.2)	0.9	.83
Intimacy	21.8 (20.4)	20.5 (25.6)	1.3	.89
Stigma	32.3 (28.2)	29.6 (28.0)	2.7	.83
Complications	29.9 (22.4)	24.7 (32.9)	5.2	.79
Sum score	35.2 (20.2)	33.2 (21.2)	2.0	.98

Abbreviations: RFIPC: rating form of IBD patient concerns, V1: baseline,V2: 6 months.

**Table 6:** Comparison of scores at V1 and V2 in patients reporting improvement or deterioration of IBD symptoms. Effect-size calculated with Cohen's *d*.

	*n*	V1	V2	Difference (95% CI)	Cohen's *d *	*P* value
Improvement of symptoms mean (SD)						
F1 Impact of disease	22	45.4 (18.9)	26.6 (17.2)	18.7 (12.6–24.9)	1.04	<0.001
F2 Expectancy	22	49.1 (22.6)	31.2 (24.5)	17.9 (8.8–27.0)	0.75	0.002
F3 Treatment	22	48.4 (24.3)	23.1 (18.6)	25.4 (15.0–35.8)	1.16	0.049
F4 Intimacy	22	32.5 (25.5)	18.7 (23.0)	13.8 (6.1–21.6)	0.56	<0.001
F5 Stigma	22	34.5 (23.1)	19.2 (17.9)	15.3 (6.4–24.2)	0.74	0.009
F6 Complications	22	33.9 (21.7)	29.8 (25.2)	4.0 (−7.9–16.0)	0.17	0.112
Sum score	22	41.3 (15.7)	24.2 (16.6)	17.1 (12.0–22.3)	1.05	<0.001
Deterioration of symptoms mean (SD)						
F1 Impact of disease	19	27.8 (16.3)	42.3 (20.8)	14.5 (20.4–8.5)	−0.77	<0.001
F2 Expectancy	19	44.8 (27.0)	51.9 (24.8)	7.1 (13.9–.3)	−0.27	<0.001
F3 Treatment	19	32.5 (22.4)	44.0 (23.8)	11.6 (22.3–.9)	−0.49	0.017
F4 Intimacy	19	25.0 (22.7)	35.6 (22.2)	10.5 (21.0–.1)	−0.47	0.018
F5 Stigma	19	20.1 (16.7)	33.9 (25.7)	13.8 (23.1–4.6)	−0.63	0.002
F6 Complications	19	29.5 (20.0)	61.7 (21.5)	32.2 (42.6–21.8)	−1.55	<0.001
Sum score	19	31.7 (17.6)	42.3 (19.3)	10.6 (16.7–4.5)	−0.57	<0.001

Abbreviations: F1–F6: rating form of IBD patient concerns, factors 1–6. V1: baseline, V2: 6-months followup.
